# Will New Drugs Replace Transplants for Chronic Lymphocytic Leukaemia?

**DOI:** 10.3390/jcm10112516

**Published:** 2021-06-07

**Authors:** Shenmiao Yang, Xiaojun Huang, Robert Peter Gale

**Affiliations:** 1 National Clinical Research Center for Hematologic Disease, Peking University Institute of Hematology, Peking University Peoples Hospital, Beijing 100044, China; huangxiaojun@bjmu.edu.cn; 2 Haematology Research Centre, Department of Immunology and Inflammation, Imperial College London, London SW7 2BX, UK; robertpetergale@gmail.com

**Keywords:** chronic lymphocytic leukemia, transplant, graft-versus-leukemia, new drugs

## Abstract

Transplants have been used to treat chronic lymphocytic leukemia (CLL) for more than 35 years. Use has been restricted to <1 percent of highly selected persons typically failing concurrent conventional therapies. As therapies of CLL have evolved, so have indications for transplantation and transplant techniques. The data that we review indicate that transplants can result in long-term leukemia-free survival in some persons but are associated with substantial transplant-related morbidity and mortality. We discuss the mechanisms underlying the anti-leukemia effects of transplants including drugs, ionizing radiations, immune-mediated mechanisms and/or a combination. We discuss prognostic and predicative covariates for transplant outcomes. Importantly, we consider whether there is presently a role of transplants in CLL and who, if anyone, is an appropriate candidate in the context of new drugs.

## 1. Introduction

Few patients, if any, with chronic lymphocytic leukemia (CLL) are cured, despite important recent therapy advances [1,2]. In this review, we consider results of transplants over four decades, compare these results with those of new CLL therapies and suggest who, if anyone, is an appropriate transplant candidate today.

## 2. Transplant Outcomes

In 1996, the European Bone Marrow Transplant Group (EBMT)/International Bone Marrow Transplant Registry (IBMTR; now CIBMTR) reported data on 54 recipients of allotransplants for advanced CLL from HLA-identical siblings following intensive pretransplant conditioning [3]. Thirty-eight achieved a hematologic remission, differently defined at that time, and 24 were alive at a median of 2 years. Three-year survival was 46% (95% confidence interval (CI), 32, 60%). Twenty-five subjects died of transplant-related complications. In another report, the German CLL Study Group presented data of reduced-intensity conditioning (RIC) transplants from HLA-matched related and unrelated donors in 90 subjects with high-risk CLL. Ten-year PFS was 34% (23, 44%). Twenty-eight subjects were measurable residual disease (MRD)-negative at 1 year post-transplant, with 24 alive and leukemia-free at >10 years, indicating at least *operational cure* of CLL in some people [4]. Reducing post-transplant immune suppression and/or giving donor lymphocyte infusions to some subjects with persistent or recurrent leukemia resulted in MRD negativity. We discuss below whether these data indicate an anti-leukemia effect of graft-versus-host disease (G*v*HD), a specific anti-leukemia effect sometimes termed graft-versus-leukemia (G*v*L) or both [5]. Other reports of transplants, mostly in persons with advanced CLL, followed. These used diverse strategies, including different pre-transplant conditioning regimens (conventional versus reduced-intensity (RIC), donors (HLA-identical versus -matched; related versus unrelated), graft sources (blood versus bone marrow versus umbilical cord blood) and pre- and post-transplant immune suppression [6,7,8,9,10]. There were no randomized controlled trials (RCTs) of any of these covariates, making it impossible to recommend a specific transplant strategy [6,7,8,9,10]. We conclude that allotransplants under diverse conditions can result in long-term leukemia-free survival (LFS) in some persons with advanced CLL (Table 1). We also caution against comparing the results of transplants performed in persons receiving chemo-immuno-therapy versus new drugs because of several confounders, including subject selection biases, different pre-transplant conditioning regimens, donors and graft types. Furthermore, there is improvement in outcomes in transplants for all diseases over the interval that we survey because of the improved prevention, diagnosis and therapy of CMV infection, acute and chronic G*v*HD and supportive care.

## 3. How Is Leukemia Controlled?

Leukemia control after an allotransplant can result from the efficacy of anti-leukemia drugs and ionizing radiations, immune-mediated anti-leukemia effects which could be non-specific (G*v*HD) or leukaemia-specific (G*v*L) or combinations. There is some long-term LFS in recipients of transplants from genetically identical twins where G*v*HD is absent and is likely the result of anti-leukemia effects of drugs and radiation [22]. Another study compared outcomes of conventional pre-transplant conditioning versus RIC [23]. RIC transplants had less TRM but a higher cumulative incidence of relapse (CIR). Intensive pre-transplant conditioning was associated with better LFS and survival in transplants before 2001 but not subsequently. The reason for this is uncertain. Conditioning regimens with and without ionizing radiations have similar TRM, CIR, progression-free survival (PFS) and survival [12]. There was a reasonably strong immune-mediated posttransplant anti-leukemia effect but researchers were unable to determine whether this resulted from G*v*HD, G*v*L or both [24]. The German CLL study group reported that some subjects previously MRD-test-positive became -test-negative after stopping post-transplant immune suppression and/or receiving a donor leukocyte infusion (DLI) [5]. Most subjects developed clinical G*v*HD consistent with a non-specific anti-leukemia effect. Another study reported that early complete T-cell chimerism correlated with a higher likelihood of becoming MRD-negative but also with higher TRM and acute G*v*HD, offsetting any clinical benefit [25].

## 4. Predicting Outcomes

There are several reports of predictive covariates for transplant outcomes in CLL [16,26]. In most studies, disease state and comorbidity index were adverse risk factors. Some studies reported that poor-risk covariates such as del (17p)/*TP53* mutation did not impact post-transplant event-free survival (EFS) [16,26,27]. The Center for International Blood and Marrow Transplant Research (CIBMTR) reported a prognostic score which also included high blood lymphocyte concentration and cytogenetic risk category (especially del(17p) and complex karyotype with ≥5 abnormalities) which correlated with poor PFS [28]. Models such as these can be used to counsel people with CLL regarding predicted transplant outcomes.

Several prognostic and predictive scores estimate if or when someone with CLL will need therapy and the survival of persons treated with new drugs (Table 2). Examples include the CLL-IPI, IPS-E, CLL1 prognostic model (CLL1-PM), BALL score, four-factor prognostic model, SRS_I_ and others, which use covariates such as age, stage, hemoglobin concentration, lactate dehydrogenase, beta-2 microglobulin and mutation states of *IGHV* and *TP53* to predict outcomes [29,30,31,32,33,34,35]. A brief response duration to a prior therapy is an adverse risk covariate regardless of therapy type [32]. Several studies correlated PFS and/or survival with negative results of MRD testing at the end of therapy in persons receiving venetoclax-based treatment or fludarabine, cyclophosphamide and rituximab (FCR) [36,37]. In summary, although it is possible to predict outcomes of cohorts of persons with CLL using a few covariates and receiving diverse therapies, accurate prediction at the individual level is difficult.

## 5. Transplant versus Current Therapies

The most important clinical question is how outcomes of transplants compare with those of current CLL therapy. In the era before new drugs, this question could only be approached indirectly because we lacked RCTs. For example, Kharfan-Dabaja and co-workers used estimates from a systematic review and data from meta-analyses to construct a Markov decision model comparing these approaches [39]. They concluded that there was better quality-adjusted life expectancy and survival with allotransplants.

Beginning in 2007, the US FDA approved 10 new anti-CLL drugs, including alemtuzumab, bendamustine, ofatumumab, rituximab, obinutuzumab, ibrutinib, idelalisib, duvelisib, venetoclax and acalabrutinib. For example, Bruton tyrosine kinase (BTK)-inhibitors such as ibrutinib and acalabrutinib improve CLL therapy, reducing the impact of adverse prognostic covariates such as fludarabine resistance, del (11q), unmutated IGHV and TP53 mutation/abnormality and purine-analogue resistance [40,41,42,43,44,45]. Therapy with a BCL2-inhibitor such as venetoclax and rituximab or obinutuzumab results in high rates of MRD negativity and good PFS [36,46]. Phosphoinostide 3-kinase (PI3K) inhibitors such as idelalisib and duvelisib are effective in persons failing prior therapies [47,48]. These advances have changed the definition of risk categories in CLL and reduced the numbers of persons classified as high-risk who might be appropriate candidates for a transplant. Between 1987 and 2010, when new drugs emerged, data on 712 allotransplants were reported to the Centre for Blood and Marrow Research (CIBMTR), or roughly 31 per year. In contrast, from 2011 to 2019, 31 allotransplants were reported, or three per year, suggesting a substantial decrease, which, of course, could be transient if new drugs are simply delaying the use of transplants (Prof. M. A. Eapen, CIBMTR and Medical College of Wisconsin). Even at the highest transplant rate and assuming substantial under-reporting, these data indicate that transplants are used in an infinitesimally small proportion of the approximately 0.5 million people with CLL in the US and EU.

It is important to consider transplants in the context of these new CLL drugs and prognostic models. Two EBMT studies reported transplant outcomes in subjects receiving ibrutinib or idelalisib [49,50]. Without a comparator arm or comparison to historical controls, and considering obvious selection biases, it is impossible to know if recent transplant outcomes differ from those reported previously. A non-randomized study with few data in subjects receiving BTK-, PI3K- or BCL-2-inhibitors pre-, peri- and/or post-transplant reported transplant outcomes similar to those reported in transplant recipients receiving conventional CLL drugs, but it is impossible to comment critically [20].

New CLL drugs can also be given to people relapsing after a transplant or to prevent post-transplant relapse. In several studies, subjects relapsing post-transplant responded to subsequent therapy with new drugs such as ibrutinib [4,51,52]. Whether giving new drugs post-transplant to prevent relapse improves outcomes is unknown [18].

Based on these data, we can make the following conclusions. (1) There are no convincing data to confirm that giving new drugs pre-transplant improves transplant outcomes. This would require data from a randomized clinical trial; no such data are available, nor is such a trial likely to be performed. The popular notion that persons with advanced CLL can receive these new drugs as a bridge to transplant is attractive but unproven. (2) Persons relapsing after a transplant respond to new drugs. (3) There are no convincing data to confirm that giving new CLL drugs post-transplant prevents relapse or improves outcomes. In summary, although there is much enthusiasm for using new CLL drugs in the context of transplants, there are presently few supporting data.

## 6. Who Should Get a Transplant Today?

Several organizations and scientific and medical bodies have published consensus statements or practice guidelines on the use of transplants and/or CAR-T-cells in persons with CLL [53,54,55,56,57]. None of these are evidence-based and we urge caution in accepting them. Some recommendations are based on comparing data from phase 1/2 trials in selected subjects with historical or otherwise matched controls. Such comparisons are scientifically and statistically flawed and often reach incorrect conclusions that are unconfirmed in phase 3 studies. Other recommendations are based on so-called *consensus* statements or practice guidelines. Elsewhere, we comment on the poor scientific validity of these metrics [58,59].

**Figure 1:**
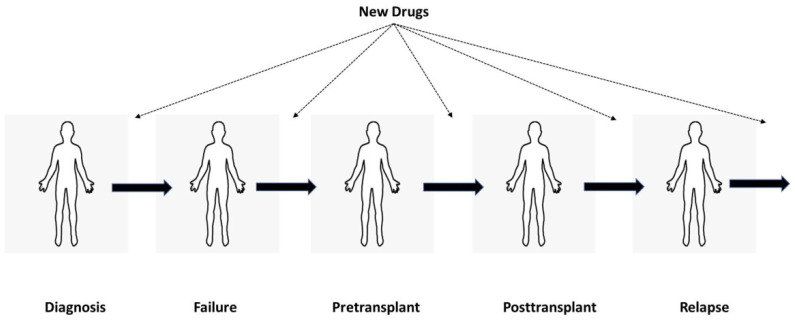
The possible place of new CLL drugs in a typical transplant scheme.

Given these limitations, how can one decide who is an appropriate transplant candidate today? Any recommendation is of course subjective in the absence of RCTs. We believe that persons unresponsive or rapidly failing ≥ 1 new therapies may be appropriate. The possible place of new CLL drugs in a typical transplant scheme is shown in Figure 1.

## 7. Summary

Transplants can *operationally cure* some persons with advanced CLL, including some failing current new therapies. We suggest that these cures result from high-dose anti-leukemia drugs, ionizing radiations and immune-mediated mechanisms, which may combine differently in different persons. Transplant outcomes seem to have improved but selection biases and other confounders discussed above make this conclusion uncertain. There are no RCTs comparing outcomes of transplants with current therapies, making the decision to perform a transplant subjective. Nevertheless, we suggest that transplants may be an appropriate intervention in some persons with CLL. When to intervene with a transplant, after failing alternative therapies or sooner, especially in young persons with advanced leukemia, is uncertain. Data from studies of chimeric antigen receptor (CAR)-T-cells in CLL are too few to evaluate critically, but this may represent another cell-based therapy of CLL with fewer adverse events compared with transplants.

## Figures and Tables

**Table 1:** Transplant outcomes in studies with ≥50 subjects.

Year	Reference	No. of Subjects	NRM (95% CI)	CIR (95% CI)	PFS/EFS (95% CI)	Survival (95% CI)
After chemo-immuno-therapy						
2013	[11]					
		32 (MAC)	48% (29, 64%)	17% (6, 33%)	36% (19, 52%)	49% (31, 65%)
		76 (RIC)	16 (9, 26%)	40% (27, 52%)	43% (31, 55%)	63% (51, 73%)
2014	[12]					
		126 (TBI)	48% (39, 57%)	17% (11, 25%)	34% (26, 43%)	42% (33, 51%)
		54 (drugs)	50% (36, 64%)	22% (11, 35%)	28% (15, 42%)	33% (19, 48%)
2017	[13]	2589	40% (37, 42%)	32% (30, 35%)	28% (25, 31%)	35% (32, 38%)
2017	[4]	100	20% (15, 36%)	46% (43, 67%)	34% (23, 44%)	51% (40, 62%)
2017	[14]	197	23% (17, 29%)	39% (32, 45%)	38% (31, 46%)	52% (44, 59%)
2018	[15]	117	44% (34, 54%)	26% (16, 35%)	30% (20, 41%)	38% (27, 49%)
2019	[16]					
		86 (NMA)	35% (NR)	28% (NR)	38% (NR)	46% (NR)
		346 (RIC)	32% (UA)	25% (NR)	43% (NR)	52% (NR)
2020	[17]	64	24% (13, 36%)	36% (23, 49%)	37% (26, 54%)	52% (40, 68%)
After new drugs						
2020	[18]	67 (19 new drugs)	28% (NR)	38% (NR)	31% (NR)	38% (NR)
2020	[19]					
		30 new drugs	7% (1, 19%)	21% (8, 38%)	72% (52, 85%)	87% (68, 95%)
		78 CIT	NR	NR	58% (46, 68%)	69% (58, 78%)
2020	[20]	65 new drugs	13% (6, 26%)	27% (17, 41%)	63% (50, 74%)	81% (70, 90%)
2020	[21]	72 idelalisib	31% (20, 43%)	25% (14, 36%)	44% (33, 58%)	59% (45, 70%)

NRM: non-relapse mortality; CIR: cumulative incidence of relapse; PFS: progression-free survival; EFS: event-free survival; MAC: myeloablative conditioning; RIC: reduced-intensity conditioning; TBI: total body irradiation; NMA: non-myeloablative; CIT: chemoimmunotherapy; NR: not reported.

**Table 2:** Prognostic and predictive scores for persons with CLL receiving new drugs.

		Co-Variates	Risk Cohort	2–3 y Survival or PFS (95% CI)
**B-ALL [32]**	New drugs	B_2_-microglobulin ≥ 5 mg/L LDH > ULN Hemoglobin < 110–120 g/L Time to failure < 2 years (Score 1 for each)	Low (score 0–1) Intermediate (score 2–3) High (score 4)	Low 90% (85, 93%) Intermediate 80% (75, 83%) High 56% (44, 66%)
**SRS_I_ [33,34]**	Ibrutinib	Hemoglobin < 110–120 g/L (score 2) B_2_-microglobulin ≥ 5 mg/L (Score 1) LDH > ULN (Score 2)	Low (score 0) Intermediate (score 1–3) High (score 3–4)	Low 95% intermediate 81% High 61%
	Idelalisib/ Rituximab			Low 95% Intermediate 81% High 61%
**4-factor** **[35]**	Ibrutinib	TP53 aberration Prior therapy B_2_-microglobulin ≥ 5 mg/L LDH > 250 U/L (Score 1 for each)	Low (score 0–1) Intermediate (score 2) High (score 3–4)	Low 93% Intermediate 83% High 63%
**MRD [38]**	Venetoclax/Rituximab	MRD	uMRD: <10^−4^ Low ≥ 10^−4^ to <10^−2^ High ≥ 10^−2^	Low 52% (32, 73%) High 8% (0, 24%)

PFS: progression-free survival; LDH: lactate dehydrogenase; ULN: upper limit normal; MRD, minimal residual disease; uMRD, undetectable MRD.
